# Macrophage-derived *Spp1* promotes intramuscular fat in dystrophic muscle

**DOI:** 10.1172/jci.insight.181946

**Published:** 2025-07-08

**Authors:** Philip K. Farahat, Chino Kumagai-Cresse, Raquel L. Aragón, Feiyang Ma, Justin K. Amakor, Alejandro Espinoza, Irina Kramerova, Robert J. Jimenez, Bradley M. Smith, Jesus Perez, Rachelle H. Crosbie, Apoorva H. Nagendra, Jackie McCourt-Towner, Gerald Coulis, Oluwatayo F. Ikotun, April D. Pyle, Matteo Pellegrini, Elizabeth M. McNally, S. Armando Villalta, Melissa J. Spencer

**Affiliations:** 1 Department of Physiology and Biophysics,; 2 Muscle Biology and Disease Research Center, and; 3 Institute for Immunology, University of California, Irvine, California, USA.; 4 Department of Neurology,; 5 Molecular Biology Institute,; 6 Department of Human Genetics,; 7 Department of Integrative Biology and Physiology,; 8 Department of Molecular and Medical Pharmacology,; 9 Eli and Edyth Broad Stem Cell Center,; 10 Department of Microbiology, Immunology and Molecular Genetics, and; 11 Department of Molecular, Cellular and Developmental Biology,UCLA, Los Angeles, California, USA.; 12 Center for Genetic Medicine, Northwestern University Feinberg School of Medicine, Chicago Illinois, USA.; 13 Department of Neurology, University of California, Irvine, California, USA.; 14 Jonsson Comprehensive Cancer Center, UCLA, Los Angeles, California, USA.

**Keywords:** Genetics, Muscle biology, Monogenic diseases, Muscle

## Abstract

Duchenne muscular dystrophy (DMD) is a progressive muscle wasting disorder involving cycles of muscle degeneration and regeneration, leading to accumulation of intramuscular fibrosis and fat. Ablation of Osteopontin/*Spp1* in a murine model of DMD (*mdx*) improves the dystrophic phenotype, but the source of Spp1 and its impact on target cells in dystrophic muscles remain unknown. In dystrophic muscles, macrophages are the predominate infiltrating leukocyte and express high levels of Spp1. We used macrophage-specific ablation combined with single-cell transcriptional profiling to uncover the impact of macrophage-derived Spp1 on cell-cell interactions in *mdx* muscles. Ablation of macrophage-specific *Spp1* (*cKO*) correlated with reduction of 2 PDGFRa^+^ stromal cell populations, expressing *Lifr^+^* and *Procr^+^*. Sorting and transcriptional profiling of these populations confirmed that they are enriched in adipogenesis genes and are highly related to fibroadipogenic precursors (FAPS). These adipogenic stromal cells (ASC) displayed more adipogenic potential in vitro compared with FAPS, likely due to a more differentiated state. Reduction of ASCs correlated with reduced intramuscular diaphragmatic fat and improved diaphragm function. These data suggest a role for myeloid-derived Spp1 in the differentiation of stromal cells towards an adipogenic fate, leading to accumulation of intramuscular fat in dystrophic muscles.

## Abstract 2

Macrophage-derived Spp1/osteopontin promotes differentiation of stromal cells towards an adipogenic fate, which correlates with accumulation of intramuscular fat and worsened function in dystrophic muscles.

## Graphical abstract

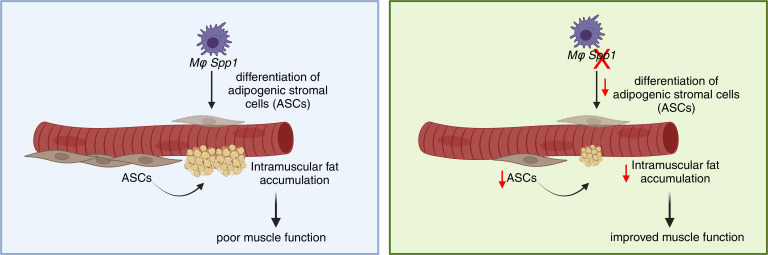

## Introduction

Duchenne muscular dystrophy (DMD) is one of the most common inherited, lethal childhood diseases. This X-linked recessive disorder is caused by mutations in the *DMD* gene encoding dystrophin (1), an important sarcolemmal membrane protein that protects myofibers from contraction-induced injury (2–4). The lack of functional dystrophin in DMD leads to chronic cycles of muscle degeneration and regeneration and accumulation of TGFb (5).

In acute muscle injury, healthy muscle repair follows a carefully orchestrated process that is initiated by infiltration of neutrophils and then Ly6c^hi^, F4/80^lo^ monocytes to the damaged area (6–8). These monocytes differentiate from a proinflammatory M1 phenotype towards a proregenerative macrophage phenotype (Ly6c^lo^, F4/80^hi^) (8–11). Progression from a proinflammatory microenvironment, induced by M1-like macrophages, to a proreparative microenvironment, induced by M2-like macrophages, is crucial for regulating stromal cell extracellular matrix secretion and growth factors that support regeneration (12, 13). This phenotypic switch is accompanied by acute activation and expansion of stromal cells, including fibroadipogenic progenitor cells (FAPs) and fibroblasts, which lay down the connective tissue that provides structural support for the damaged site (13). Infiltrating myeloid cells are also essential for activation of muscle stem cells (MuSC) that regenerate myofibers (12, 14, 15). The microenvironment established by this precisely regulated inflammatory and stromal cell response is crucial for proper muscle regeneration.

In the setting of chronic damage and repair, such as in DMD muscles, a dysregulated inflammatory response leads to the prolonged presence of both proinflammatory and proregenerative macrophages and accumulation of the profibrotic cytokine, TGF-β (5, 16). While evidence of M1 and M2 macrophage activation has been documented in dystrophic muscle (8, 9) more recent studies have challenged the view that the M1 and M2 paradigm accurately reflects the molecular phenotype and function of dystrophic muscle macrophages. Single-cell transcriptomic studies showed that skeletal muscle resident macrophages and recruited monocytes acquire a molecular signature characterized by high expression of *Lgals3*, *Spp1*, *Trem2*, *Gpnmb*, and *Cd9*, none of which are classic markers of M1 or M2 activation (11). This dysregulated macrophage state, combined with profibrotic signaling through TGF-β, hinders muscle regeneration while promoting muscle fibrosis and accumulation of intramuscular adipose tissue (IMAT) (17–19). The source of IMAT has been previously attributed to FAPS (12–14).

We previously identified osteopontin (Spp1) as a modifier of TGF-β1 expression that promotes dystrophic disease in *mdx* muscle (6, 8, 20, 21). Spp1 is a ubiquitously expressed, matricellular protein that is highly upregulated in dystrophic muscle (6). Global knockout of *Spp1* in *mdx* mice led to an improved dystrophic phenotype, including increased muscle function, increased muscle regeneration, reduced proinflammatory (M1) muscle macrophages, reduced *TGF-*β*1* expression, and reduced collagen deposition, compared with *Spp1-*sufficient *mdx* mice (6, 8, 20, 21).

Spp1 is a ubiquitously expressed protein that can be secreted or reside intracellularly. It is involved in a diverse array of biological processes and signaling pathways. In the extracellular matrix, Spp1 engages multiple cell surface receptors such as integrins, CD44, and components of the ECM such as heparin (22, 23). In the dystrophic niche, Spp1 is expressed by a variety of cell types, including immune cells, stromal cells, muscle fibers, endothelial cells, and muscle stem cells (24). Importantly, Spp1 is posttranslationally modified by phosphorylation, glycosylation, and transglutamination and is cleaved by thrombin and metalloproteases. Thus, depending on the cellular source of Spp1, its posttranslational modifications, and the proximity of receptors, the effects on target cells can vary. However, the influence of cell-specific Spp1 on different target cells within the highly dynamic dystrophic niche remains poorly understood.

Here, we generated myeloid-specific conditional knockouts of *Spp1* (*cKO*) and used scRNA-seq to evaluate how myeloid cell–derived Spp1 impacts the cellular milieu of dystrophic skeletal muscles. We demonstrated that cKO muscles showed reduction of a stromal cell subpopulation that is highly adipogenic, referred to as adipogenic stromal cells (ASCs). ASCs are highly related to FAPS but show an enhanced lipid profile. These changes also correlate with reduced intramuscular fat and improved muscle function. Therefore, myeloid-derived Spp1 contributes to fat accumulation by promoting differentiation of stromal cells towards an adipogenic fate and contributing to worsened dystrophic disease.

## Results

### Myeloid cell–derived Spp1 promotes immune cell infiltration of neutrophils, T and B cells, and has the opposite effect on macrophages in dystrophic muscles.

**Figure 1:**
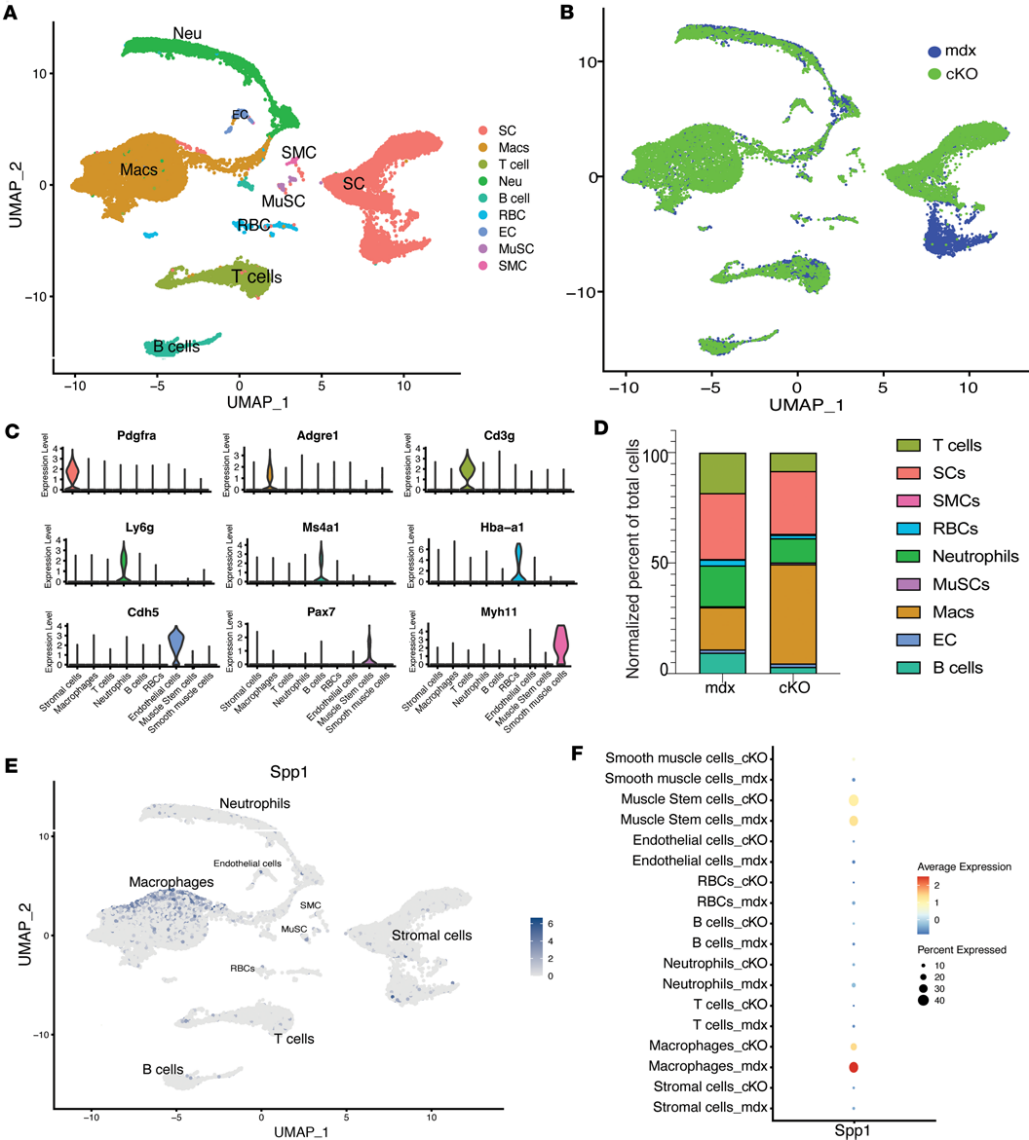
scRNAseq of muscle-infiltrating mononuclear cells suggests cross talk between myeloid-derived Spp1 and a subpopulation of Pdgfra^+^ stromal cells. Mononuclear cells isolated from mdx and cKO muscles were subjected to scRNAseq followed by informatic analysis. (**A**) UMAP plot shows unsupervised clustering of mononucleated cells isolated from skeletal muscles of 12-week-old mice (*n* = 3 per genotype). (**B**) Dim plot of cKO (green) and mdx (blue) cells. The total number of cells isolated from each genotype is shown in the lower right of the plot. (**C**) Violin plots of marker genes used to facilitate cell type annotation. (**D**) Graph of the relative cell type proportions, shown by genotype, of each cell type normalized to total cells in the sample. Colors of bars correspond to colors of cell types in **A**. (**E**) Feature plots of *Spp1* expression (dark blue) across all cell types shown in the UMAP (gray). (**F**) Dot plot showing *Spp1* expression in major cell types from cKO muscle compared to mdx.

We generated myeloid specific, *Spp1* conditional knockout mice (*cKO*) and assessed cellular profiles using single cell RNA-seq (scRNAseq) to determine how myeloid cell–derived *Spp1* impacts cellular profiles within dystrophic muscles (Supplemental Figure 1 shows the construct used to generate the mice; supplemental material available online with this article; https://doi.org/10.1172/jci.insight.181946DS1). Mononucleated cells were isolated from limb and diaphragm muscle of 3-month-old cKO (*n* = 3) and mdx control (*n* = 3) mice followed by scRNAseq using the 10x Genomics platform. We analyzed 10,657 cells from cKO muscle and 7,156 cells from mdx muscle, revealing nine major cell types (Figure 1A), including stromal cells and immune cells such as macrophages, neutrophils, T cells and B cells (Figure 1C). Macrophages are the primary source of Spp1 in dystrophic muscle (Figure 1, E and F). Lyz2Cre primarily impacted Spp1 in macrophages (Figure 1F).

We further analyzed how cellular frequencies changed by myeloid-specific deletion of Spp1 (Figure 1D). Our prior studies showed that global *Spp1* knockdown correlated with a significant decrease in neutrophils in 4-week-old muscle (8). Ablation of myeloid-derived *Spp1* resulted in a decreased frequency of neutrophils (NP), as well as reductions in the frequencies of T and B cells (Figure 1B). Intriguingly, an increase in the proportion of macrophages was observed in the cKO, which could be due to an autocrine effect of *Spp1* on macrophage survival, proliferation, or infiltration, or to concomitant decreases in other immune cell populations. While muscle stem cells were not well represented in the single-cell data set, they were identified by the marker Pax7 (Figure 1C) and were shown to also have high Spp1 expression (Figure 1F). It should be noted that these cells are underrepresented in the current data set because the cell isolation protocol was not optimized for recovering muscle stem cells. Additionally, Spp1 expression is observed in several immune cell types and stromal cells, consistent with previous reports in the literature. When UMAPs were viewed by genotype, there was a striking absence of one stromal cell type in cKO compared with mdx (Figure 1D); however, no difference in the overall percentage of stromal cells was observed in the 2 genotypes (Figure 1C).

### Myeloid-specific deletion of Spp1 does not dramatically impact monocyte subtypes.

**Figure 2:**
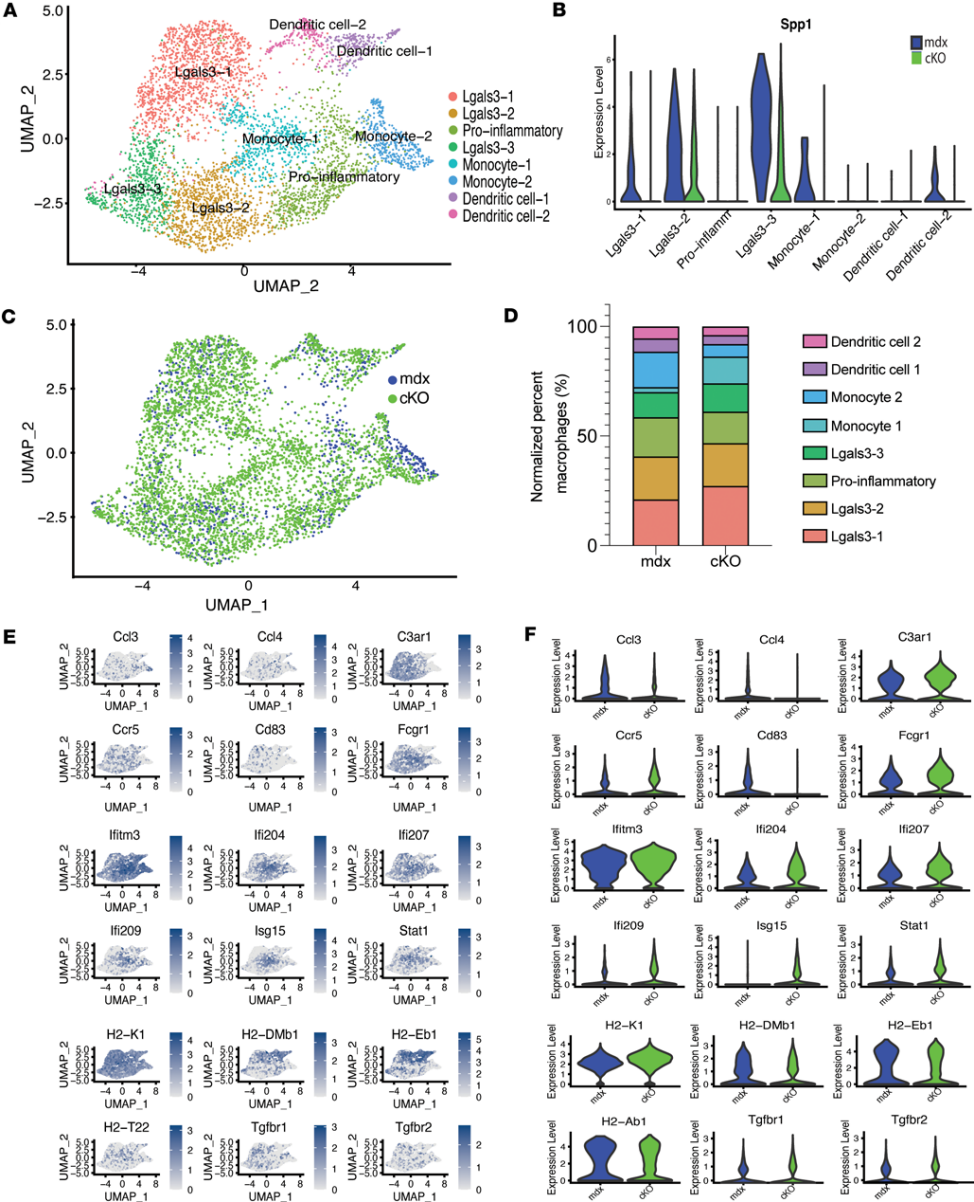
Monocytic cells show few phenotypic changes. (**A**) UMAP plot shows unsupervised subclustering of monocyte subpopulations from mdx and cKO. (**B**) Violin plot shows the levels of *Spp1* by each monocytic subtype in mdx (blue) and cKO (green). (**C**) Dim plot shows the distribution of monocytic subpopulations by genotype (mdx, blue; cKO, green). (**D**) Proportion of cells in each subpopulation by genotype (normalized to total monocytic cells in each sample). (**E**) Feature plots show genes from single cell RNA sequencing data (blue) projected on the monocyte subcluster (gray) showing genes that defined cell phenotypes. (**F**) Violin plots from single cell RNA sequencing data showing significantly different macrophage genes by genotype (mdx, blue; cKO, green).

To determine the impact of myeloid cell–derived *Spp1* on monocyte phenotypes, we conducted unsupervised subclustering of monocytes and identified 8 distinct subtypes: 2 monocyte populations, 4 macrophage populations, and 2 dendritic cell populations (Figure 2A and Supplemental Figure 2). Markers that define monocyte phenotypes from mdx and cKO are shown in Figure 2E. Monocyte clusters 1 and 2 were marked by *Sell, Isg15,* and *Ifitm6* expression. Three macrophage populations expressed high *Lgals3* and were referred to as Lgals3-1, Lgals3-2, and Lgals3-3 and were further defined by high *Apoe*, *Cd63*, and *Spp1*. Dendritic cells expressed high MHCII genes like *H2-Dmb1* and *H2-Oa*. Lgals3 clusters 2 and 3 expressed the highest *Spp1*. All Spp1-expressing clusters showed a drastic reduction in Spp1 in the cKO (Figure 2B, compare blue and green) (8). Lgals3-2 cells also expressed *Arg1* while Lgals3-3 expressed *Igf1* (11). The proportion of different monocyte/macrophage subtypes was evaluated in the 2 genotypes (Figure 2, C and D). While the proportion of Lgals3-3 and Lgals3-2 remained similar in the 2 genotypes, a mild increase in Lgals3-1 was observed in the cKO, while both monocyte subclusters slightly decreased in the cKO (Figure 2C). Although the overall number of macrophages increased in the cKO (mdx:969 vs cKO 4,028), cKO macrophages did not show large changes in the cellular frequency of subtypes (Figure 2D). Inflammatory genes were either unchanged or slightly reduced in the cKO, including the chemokines *Ccl3* and *Ccl4* (Figure 2F) and *Tgfb1* (Supplemental Figure 2B). However, IFN genes such as *Ifi207*, *Ifi204*, and *Isg15* were slightly increased in cKO monocytes (Figure 2F).

### Reduced abundance of 2 stromal cell subpopulations in cKO.

**Figure 3:**
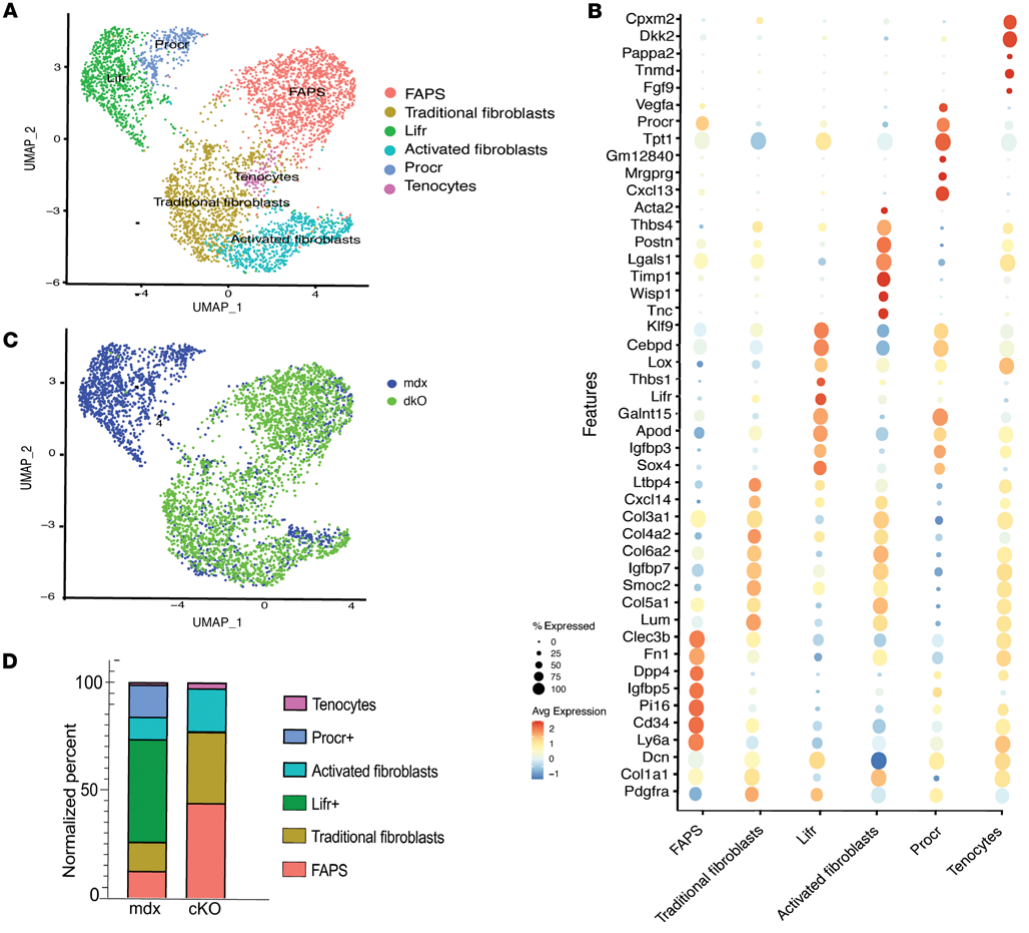
Stromal cell subclustering reveals reduction of 2 stromal cell subpopulations in cKO muscles. (**A**) UMAP plot shows unsupervised clustering of Pdgfra^+^ stromal cell subpopulations from cKO and mdx skeletal muscle. (**B**) Dotplot of top cluster markers used to identify stromal cell subtypes in **A**. (**C**) Dim plot by genotype (mdx, blue; cKO, green). Overall cell numbers for each genotype are shown in the lower right corner of the graph. (**D**) Proportion of stromal cell subtypes in each genotype, normalized to the number of total Pdgfra^+^ cells in each sample.

To further query stromal cell phenotypes, all *Pdgfra^+^* cells were subclustered, leading to the identification of 6 transcriptionally distinct clusters (Figure 3A). Markers that define these subclusters are shown in Figure 3B and Supplemental Figure 3. FAPs were marked by *Ly6a* (SCA1) and *Cd34*; traditional fibroblasts were enriched in collagen genes such as *Col3a1*, *Col4a2*, and *Col6a2* and showed the highest expression of *Pdgfra;* and an activated fibroblast population was identified by expression of *Postn* and *Acta2*. A small *Tnmd*(+) tenocyte population was also identified. Two subpopulations of stromal cells, which we call *Lifr*^+^ and *Procr*^+^ subclustered separately from FAPS and fibroblasts (Figure 3, A and C) and were reduced in the cKO compared to mdx (Figure 3, C and D), and FAPs, traditional fibroblasts and activated fibroblasts were proportionally increased in the cKO (Figure 3D). In fact, the number of cells derived from the cKO was 2,591, while cells from mdx was 1,896. Stromal cells exhibit low levels of *Spp1* expression, and its deletion via *Lyz2*-Cre did not result in a reduction of *Spp1* (Supplemental Figure 4). The proportional expansion of fibroblasts and FAPs in the cKO was accompanied by upregulation of extracellular matrix (ECM) genes, including *Col1a1*, *Col8a1*, *Col3a1*, and *Col5a1*, as well as increased expression of *Spp1* receptors (Supplemental Figure 3 and Supplemental Figure 4, C and D). Despite these molecular and cellular changes, there was no corresponding increase in fibrosis, as assessed by morphology and hydroxyproline content (Supplemental Figures 4 and 8). These alterations in stromal cell composition suggest a role for myeloid-derived *Spp1* in supporting the maintenance of *Procr*^+^ and *Lifr*^+^ populations. However, while prior studies have linked *Spp1* to fibrosis in dystrophic muscle (6, 8, 20, 21), the profound shifts observed in stromal subtypes towards fibroblasts and FAPS in the cKO complicate the ability to definitively determine the role of myeloid-derived *Spp1* in fibrosis progression.

### Isolation of Lifr^+^ and Procr^+^ stromal cells reveals an enhanced adipogenic gene expression profile.

**Figure 4:**
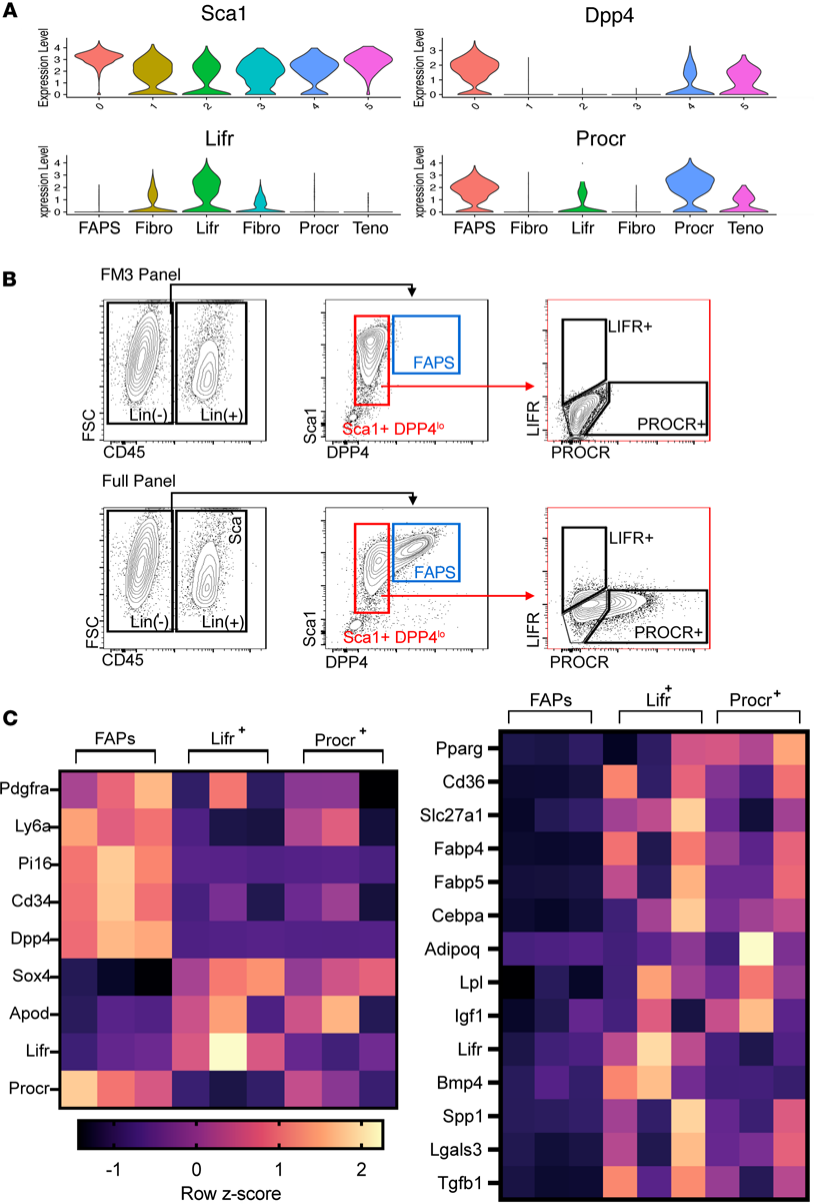
Sorted Lifr^+^ and Procr^+^ stromal cells demonstrate an enhanced adipogenic profile compared with FAPS. (**A**) Violin plot of markers identified in single cell RNA sequencing data that were used to design the sorting strategy used in **B**. (**B**) Representative plots showing the steps involved in isolation of Lifr^+^ and Procr^+^ populations from muscles. The “FM3” panel shows controls and the “Full panel” shows sorted cells. After gating on Lin^–^ cells (far left panel), FAPS were isolated as Sca1^+^ DPP4^+^ and further as Lifr^–^ Procr^+^. Lifr^+^ and Procr^+^ cells were isolated after gating on Sca1^med^ DPP4^med^ and then evaluated for expression of Lifr and Procr. (**C**) (left) Heatmap of z-scores generated from bulk RNAseq of sorted stromal cells showing expression levels of key cluster markers in FAPs, Lifr^+^, and Procr^+^ cells isolated from skeletal muscle of mdx mice (*n* = 3). (right) Heatmap of z-scores generated from bulk RNAseq of stromal cells showing expression levels of adipogenic genes (*n* = 3). Note the enrichment of adipogenesis genes in Lifr^+^ and Procr^+^ cells (ASCs) compared with FAPs.

To confirm that *Lifr*^+^ and *Procr*^+^ cells are a distinct stromal cell population in vivo, we designed a FACS sorting strategy guided by markers in the scRNAseq dataset (Figure 4, A and B). The sorting strategy used antibodies to surface proteins Sca1 (*Ly6a*) and Dpp4 to discriminate FAPS from *Lifr*^+^ and *Procr*^+^ cells. Dpp4 is most highly expressed in FAPs (Figure 4B), while *Lifr*^+^ and *Procr*^+^ cells were Sca1^med^/Dpp4^med^ and were further sorted by Lifr and Procr (Figure 4B). Bulk RNAseq analysis of the sorted cells support that they are *Pdgfra*^+^ stromal cells with a distinct gene expression pattern of markers that were identified in the single cell sequencing dataset (Figure 4C, heatmap of marker genes). For example, *Pi16* and *Cd34* are high in isolated FAPs, whereas Lifr^+^ and Procr^+^ cells showed higher expression of *Sox4* and *Apod* (Figure 4C). The ability to sort these cells and validate markers identified by scRNAseq suggests that these cells represent a population of stromal cells that are highly adipogenic.

To further characterize the phenotype of Lifr^+^ and Procr^+^ subpopulations, we carried out gene set enrichment analysis using markers from the scRNAseq data set (Supplemental Figure 5). This analysis revealed that both the Lifr^+^ and Procr^+^ cells are enriched in adipogenesis genes such as *Cebpd*, a master regulator of adipogenesis, and *Lifr* and *Fabp4*, which have been shown to be upregulated during adipocyte differentiation (25–28). Analysis of significant differentially expressed genes by bulk RNAseq from sorted Lifr^+^ and Procr^+^ stromal cell populations validated the increased expression of adipogenesis markers in these stromal cell clusters compared with FAPs (Figure 4C). Lifr^+^ and Procr^+^ cells also showed enrichment of genes involved in TGF-β and IGF-1 signaling, which is also reflected in the gene set enrichment analysis. Therefore, from this point forward, these cells are referred to as adipogenic stromal cells (ASCs).

### ASCs exhibit increased adipogenic activity in vitro.

**Figure 5:**
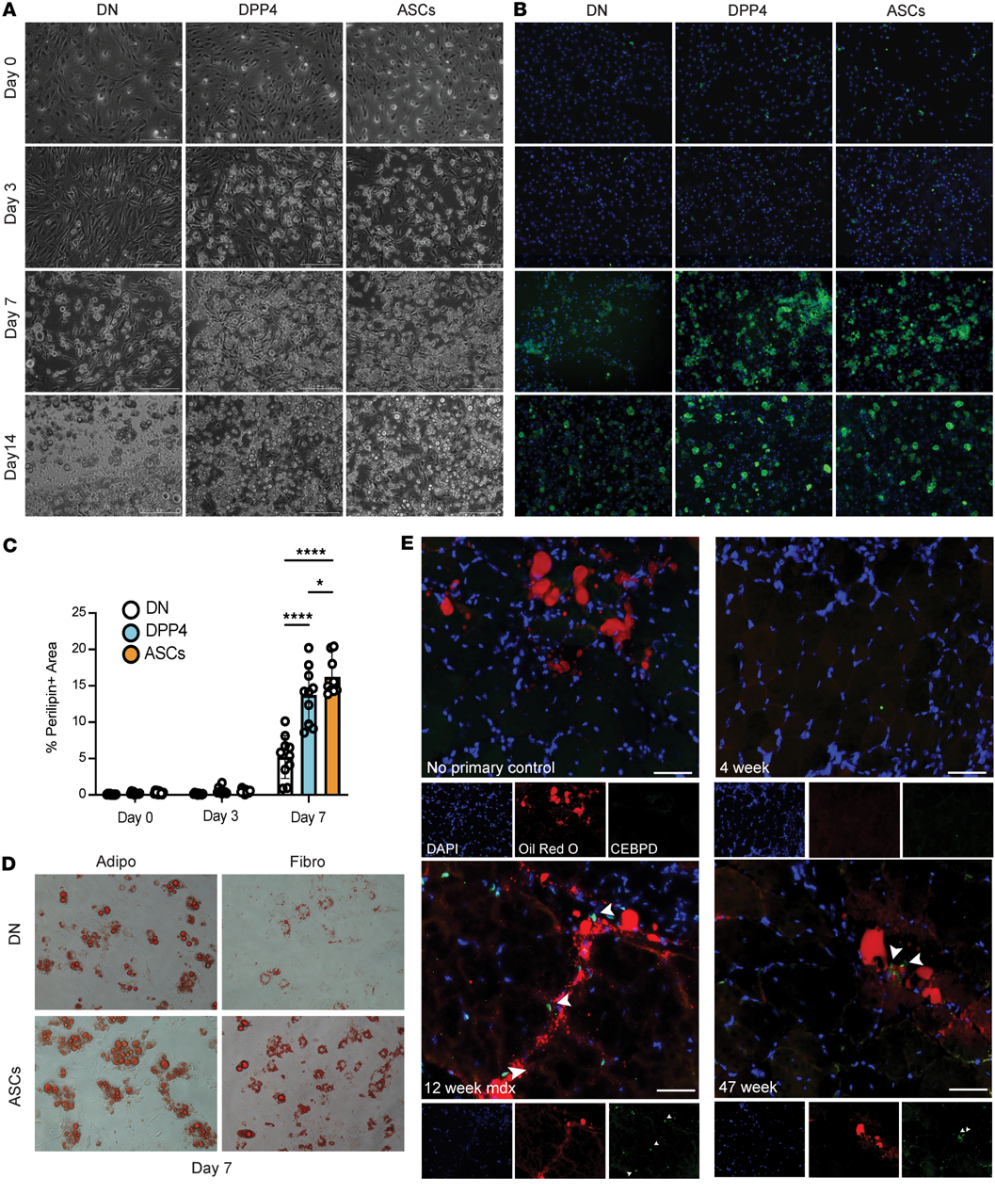
Lifr^+^/Procr^+^ stromal cell subpopulations demonstrate increased propensity for adipogenesis in vitro compared with FAPS. (**A**) Phase contrast images (**B**) and perilipin fluorescence imaging of FACS-sorted stromal cell subpopulations on days 0, 3, 7, and 14 of adipogenic differentiation. The double negative (DN) population consists of Pdgfra^+^SCA1^+^DPP4^–^PROCR^–^LIFR^–^ cells. The DPP4^+^ population is comprised of Pdgfra^+^SCA1^+^DPP4^+^PROCR^–^LIFR^–^ cells, and PROCR^+^ FAPs are Pdgfra^+^SCA1^+^DPP4^–^PROCR^+^LIFR^–^ cells. Scale bar: 200 µm. (**C**) Quantification of Perilipin^+^ area in cultured cells; 10 representative images were quantified for each timepoint. *P = 0.0175, ****P < 0.0001. (**D**) Oil Red O staining of DN and PROCR^+^ FAPs after 7 days of adipogenic or fibrogenic differentiation. (**E**) Oli Red O and CEBPD staining of 4-,12-, and 47-week-old mdx quadriceps. Arrows showing CEBPD positive cells in FITC while Oil red O fluoresces in the red channel. Scale bar: 50 μm. Statistics used include 1-way ANOVA with a post hoc Tukey’s multiple comparisons test.

To assess their adipogenic potential, ASCs and FAPS were FACS sorted and cultured under adipogenic or fibrogenic conditions (Figure 5, A and B). After 7 days of adipogenic differentiation, ASCs demonstrated a higher propensity to differentiate into adipocytes, as visualized by perilipin staining (Figure 5C). Even in fibrogenic conditions, ASCs had a higher propensity to differentiate into lipid-containing (oil-red^+^) adipocytes compared with double negative cells (Figure 5D). Additionally, an accumulation of CEBPD-positive cells was observed near Oil Red O–positive areas in the quadriceps of 12-week and 47-week-old mdx mice. Oil Red O stains neutral triglyceride and lipid droplets, which are often associated with adipocytes (Figure 5E). Conversely, in the quadriceps of 4-week-old mdx mice, there was minimal-to-no Oil Red O staining, and CEBPD-positive cells were absent. These findings suggest that ASCs likely represent a more differentiated state within the FAP lineage, contributing to adipose tissue accumulation in dystrophic muscle.

### Macrophage cKO mice show reduced intramuscular fat deposition.

**Figure 6:**
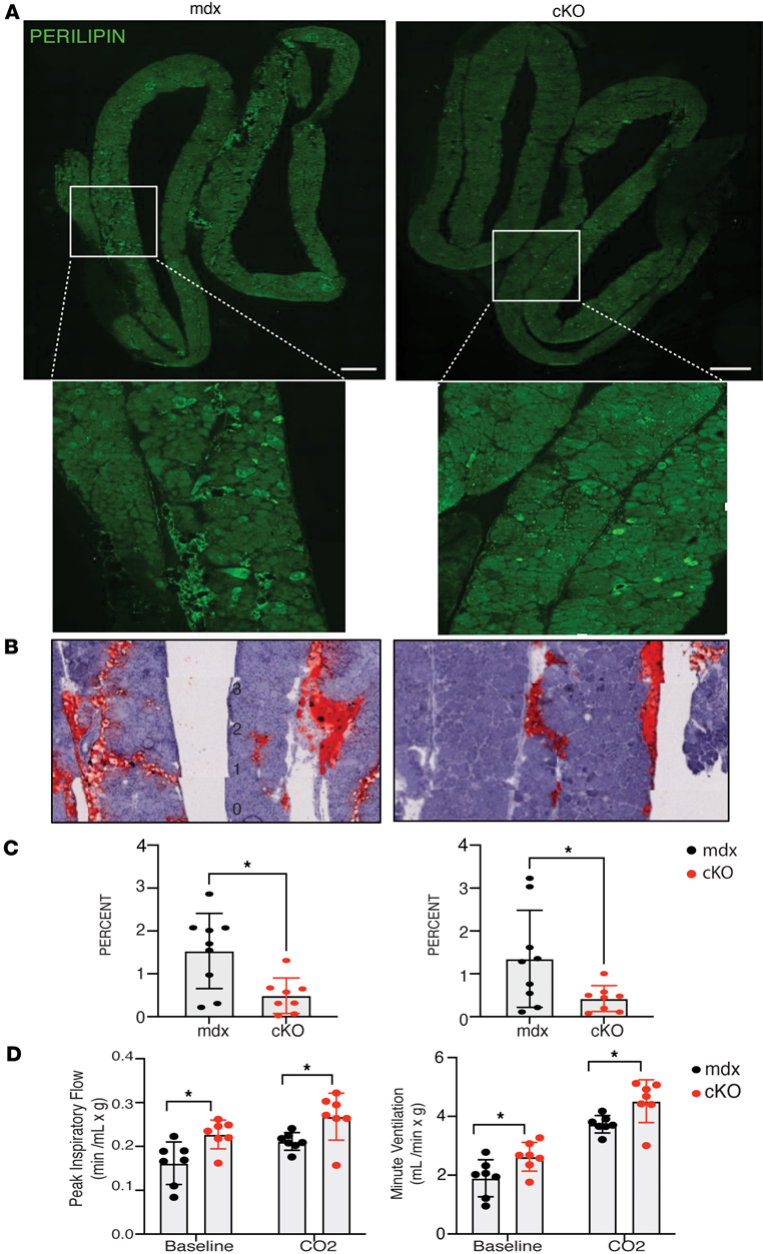
Intramuscular fat is reduced in cKO dystrophic muscles. (**A**) Immunostaining of perilipin in diaphragms from 6-month-old mdx (left) and cKO mdx muscles (right). The white box shown in the top micrographs is enlarged in the panel below. Note the presence of mature adipocytes in both genotypes of dystrophic mice. Scale bar: 500 μm. (**B**) Oil Red O stain of 6-month-old mdx (left) and cKO (right) diaphragms. The full diaphragm section can be viewed in Supplemental Figure 6, where a black box denotes the area used. (**C**) Quantification of perilipin (left) and Oil red O staining (right) normalized to cross-sectional area in diaphragms from mdx (black) and cKO/mdx (red) 3–6-month-old mice. Each dot represents 2 sections collected and averaged from each mouse. (**D**) Plethysmography to assess pulmonary function in live mice. Left graph shows increased peak inspiratory flow at baseline and in response to a hypercapnic (CO_2_) challenge in cKO mice (red) compared with mdx mice (black). Right graph shows increased minute ventilation in cKO compared with mdx. Each dot represents a single mouse (*n* = 7 each genotype). Statistics used included a 2-tailed *t*-test.

We assessed whether a decrease in ASCs was associated with reduced IMAT accumulation. While Duchenne muscles often show high levels of IMAT, the mdx mouse model of DMD is milder and fat is not a prominent feature, especially in hindlimb muscles (2, 29–32). However, the mdx diaphragm is viewed as a muscle that more closely resembles the progressive nature of Duchenne muscles (33, 34). We looked for evidence of IMAT in diaphragm muscles from 3–6-month-old cKO and mdx mice by staining for perilipin, a marker of mature adipocytes (Figure 6A) and for Oil Red O, (Figure 6B and Supplemental Figures 6 and 7) which identifies neutral triglyceride and lipid droplets often associated with adipocytes. Both mouse genotypes exhibited IMAT deposition in the diaphragm, as indicated by multiple markers, with a notable reduction observed in the cKO mice. Quantification of perilipin and Oil Red O staining in diaphragms revealed a significant decrease in perilipin-positive areas in cKO diaphragms compared with mdx controls (Figure 6C). Similarly, Oil Red O staining demonstrated a marked reduction in lipid accumulation in cKO diaphragms relative to mdx counterparts (Figure 6C). Supplemental Figure 6 shows stitched images of Oil Red O–stained diaphragms, with blue boxes highlighting the regions depicted in Figure 6.

Although Oil Red O–positive regions were also detected in the gastrocnemius and quadriceps muscles (Supplemental Figure 7), fat accumulation was substantially lower, consistent with the milder phenotype observed in mdx hindlimb muscles. These findings suggest that ASCs contribute to IMAT accumulation in the more severely affected diaphragm of dystrophic mice. Importantly, the minimal fat deposition in hindlimb muscles does not limit their suitability for assessing cellular phenotypes in the single-cell sequencing, as IMAT represents a late-stage feature, whereas the cellular phenotypes emerge earlier in the disease timeline.

To determine whether reduced IMAT correlates with functional improvements in the diaphragm, pulmonary function tests were performed. Whole-body plethysmography was conducted at baseline and following a hypercapnic challenge to stimulate respiratory drive. Results showed that cKO mice exhibited improved pulmonary function, evidenced by enhanced peak inspiratory flow, a direct indicator of diaphragm strength, and increased minute ventilation, reflecting overall respiratory performance (Figure 6D). These findings suggest a role for myeloid cell–derived Spp1 in promoting fat accumulation in dystrophic muscles and indicate a relationship between IMAT presence and muscle function in dystrophic conditions.

## Discussion

In this study, we conducted an unbiased scRNAseq transcriptomic analysis of dystrophic muscle to define the impact of myeloid cell–specific Spp1 on cell-cell interactions in the niche. We found that Spp1 from myeloid cells cross talks with stromal cells and promotes an enhanced adipogenic stromal cell state that contributes to intramuscular fat in dystrophic muscles. Myeloid cell–derived Spp1 contributes to enhanced adipogenic gene expression in these ASCs compared with FAPs, although ASCs likely represent a more differentiated form of FAPS.

Most investigations of FAPs in muscle have isolated them by sorting on PDGFRA^+^, a pan marker for stromal cells, or sorting for Lin^–^ (CD45^–^CD31^–^ITGA7^–^) SCA1^+^CD34^+^ cells (15, 28, 35). These sorted FAPS have been shown to alter myofiber regeneration, fibrosis, and IMAT accumulation (15, 34, 36, 37). Here, we demonstrated that ASCs (Lifr^+^ and Procr^+^ populations) coisolate with FAPs using the sorting strategy described above, but when further sorted for specific markers identified by our scRNAseq data set, show a more adipogenic transcriptional profile. The identification, isolation, and characterization of ASCs demonstrates that a spectrum of stromal cell subtypes exist.

Accumulation of IMAT is known to correlate with age and clinical performance in DMD (38–41) and it has been assumed that IMAT develops from the reprogramming of FAPs by the microenvironment, directing them towards an adipogenic fate. Our data suggest that Spp1 contributes to the differentiation of ASCs into adipocytes and is at least partially responsible for fat accumulation in dystrophic muscles. We also observed a correlation between increased IMAT and impaired muscle function in the diaphragm, the one muscle in mdxB10 mice that undergoes progressive degeneration and exhibits greater fibrosis than the hindlimb muscles (Supplemental Figure 9). While these cells may be a separate, distinct stromal cell subtype rather than a transient FAP differentiation cell state towards an adipogenic fate, it is more likely that ASCs derive from FAPS, possibly representing a more differentiated state, which may suggest a role for Spp1 in the adipogenic differentiation of FAPS. Lineage tracing analysis is needed to more finely define the origin of these stromal cell subtypes.

This study also identifies a role for myeloid cell–derived Spp1 in cross-talk with stromal cells. Crosstalk has been previously observed between macrophages and stromal cells in the context of acute muscle injury, a process that is carefully orchestrated to ensure proper muscle regeneration (16, 19, 42). Although ablation of myeloid-derived Spp1 was associated with increased numbers of several stromal cell populations, including FAPs, traditional fibroblasts, and activated fibroblasts, and with upregulation of ECM-related genes, this change did not lead to an overall increase in collagen content. This discrepancy may reflect the observed shifts in stromal cell composition that occurred in the conditional knockout. Nonetheless, our findings support a role for myeloid-derived Spp1 in directing stromal cell differentiation toward an adipogenic phenotype and demonstrate a correlation between IMAT accumulation and impaired diaphragm function in dystrophic muscle. These results suggest that targeting myeloid-derived Spp1 may offer a therapeutic strategy to limit adipogenic remodeling and preserve muscle function in dystrophic conditions.

## Methods

### Sex as a biological variable.

Our study exclusively examined male mice because the disease under study is Duchenne muscular dystrophy, which is an X-linked recessive disorder.

### Generation of Spp1 cKO mice.

Floxed mice (*Spp1 fl/fl* mice) were generated by flanking exons 2 and 3 of *Spp1* with LoxP sites, which removes the start sites for both intracellular and secreted forms of Spp1 after Cre-mediated recombination (Supplemental Figure 1). To achieve myeloid cell–specific deletion of Spp1, we crossed *Spp1 fl/fl* mice to *Lyz2^cre^* (also known as *LyzM^cre^*) mice (Jackson Labs) (43). The mice were made congenic to the *mdx* BL/10 background by backcrossing at least 5 times.

The *Spp1* targeting vector was made using accession #NT_109320.5 (28,332,597-28,344,133), using an 11.5 kb Fragment from a HpaI-ApaI digestion of BAC clone RP24-170A (BAC PAC Resource Center) that was cloned into pBlueScript II SK(+) and inserted into the ApaI-EcoRV site. LoxP sites were inserted after exon 1 and before exon 4 of the *Spp1* locus to engineer removal of exons 2 and 3 and generate a premature stop codon.

### Generation of Spp1 floxed dystrophic mice.

The targeting vector used to place LoxP sites flanking exons 2 and 3 of *Spp1* was electroporated into ES cells of the C57BL/6N strain by the Mouse Biology Program at the University of California Davis, Davis, California. Neomycin-resistant cells were selected and microinjected into blastocysts of the Balb/C strain. Mice with germline transmission were bred with FLP mice (C57BL/6N-Tg(CAG-Flpo)1Afst/Mmucd, provided from the Mutated Mouse Resource & Research Center at University of California, Davis). Pups in which the neomycin gene was excised were selected and bred. *Spp1-*floxed mice were crossed to C57BL/10ScSn-Dmd^mdx^/J (Jax #001801) mice that were obtained by The Jackson Laboratories.

### Crossing of Spp1-floxed mice with tissue-specific Cre-mdx mice.

*Lyz2^cre^* (B6.129P2-Lyz2^tm1(cre)lfo^/J, Jax #: 004781) mice with myeloid-specific Cre were obtained from The Jackson Laboratories and crossed to *Spp1*-floxed *mdx* mice.

### PCR for genotyping myeloid-specific Spp1 cKO mice.

PCR genotyping of Lyz2^cre^ mice used the following primers from the Jackson Labs website: Lyz2 mutant: 5′- CCC AGA AAT GCC AGA TTA CG-3′; Lyz2 common: 5′ CTT GGG CTG CCA GAA TTT CTC-3′; Lyz2 wild type: 5′-TTA CAG TCG GCC AGG CTG AC-3′. PCR for the floxed *Spp1* allele used the following primers: Forward: 5′- GGA CCT TGA GTG ACT GGT TCT-3′; Reverse: 5′- TGG ACC TGA ACT CTG TGT GC-3′.

### Mononuclear cell isolation from muscle for scRNAseq.

Mice were sacrificed and muscles from forelimbs, hindlimbs (triceps, quadriceps, tibialis anterior, gastrocnemius), and diaphragm were pooled and placed into a Petri dish containing sterile PBS (with Ca^2+^, Mg^2+^) for weighing. Muscles were washed in PBS (with Ca^2+^, Mg^2+^) and then minced in a 1:5 (weight:volume) solution of 6 mg/mL collagenase type 2 (Worthington) and 20,000 units/mL. Muscles were enzymatically digested at 37°C. Digested tissue was filtered through a 70 µm nylon filter and was washed with Ca^2+^, Mg^2+^–free PBS. Cells were pelleted by centrifugation at (330*g*) for 5 minutes. After discarding the supernatant and breaking up the pellet with 5 mL of Ca^2+^, Mg^2+^–free PBS, the conical tube was filled with Ca^2+^, Mg^2+^–free PBS and centrifuged again for 10 minutes followed by using a Cell Debris Removal kit (Miltenyi Biotech) and centrifugation at (300*g*) at 4°C. After removing the PBS and interphase (cell debris) layers, cells were washed with Ca^2+^, Mg^2+^–free PBS and centrifuged again at (3,000*g*) at 4°C. Cells were resuspended in ACK lysis buffer (Lonza) to lyse red blood cells and centrifuged at (300*g*) at 4°C for 10 minutes. The pellet was resuspended in Ca^2+^, Mg^2+^–free PBS and cells were filtered with a 40 µm nylon filter and washed with additional Ca^2+^, Mg^2+^–free PBS. After centrifugation (300*g*) at 4°C, we used a Dead Cell Removal Kit (Miltenyi Biotech). The purified cell solution was centrifuged at (300*g*) at 4°C. We discarded the supernatant and resuspended the cell pellet in a 0.04% BSA solution. To count cells, a small volume of sample was mixed 1:1 with 0.4% Trypan blue solution and counted using a hemocytometer.

### scRNAseq library construction and analysis.

scRNAseq was conducted on mononucleated cells isolated from murine skeletal muscle, as described above. Libraries were constructed using the Chromium Single Cell 3′Reagent Kits v3 (10X Genomics) at the UCLA Technology Center for Genomics & Bioinformatics (TCGB). cDNA libraries were sequenced on the NextSeq500 High Output platform to achieve approximately 20,000 reads/cell. Sequencing reads were processed using the 10X Genomics Cell Ranger (Seurat version 3.0) (44). Low-quality cells were removed when more than 20% of the UMIs were derived from mitochondrial genes, when less than 200 features and more than 1,800 unique features were detected. The data were then normalized using the default NormalizeData function parameters, the FindVariableFeatures function to select 2,000 genes with the highest standardized variance, and the ScaleData function to perform z-score transformation. We then analyzed the integrated samples by using RunUMAP, FindNeighbors, and FindClusters functions for uniform manifold approximation and projection (UMAP) visualization. The clusters were manually annotated using the top 50 differentially expressed genes produced by the FindAllMarkers function. For subcluster analysis of stromal cells and macrophages, we used the subset function in combination with the same methods discussed to analyze the integrated data.

### Gene set enrichment analysis.

Gene set enrichment analysis was performed with the WEB-based Gene Set Analysis Tool Kit (https://www.webgestalt.org/) using the Kyoto Encyclopedia of Genes and Genomes (KEGG) database using differentially expressed genes.

### Flow sorting for stromal cell populations.

Stromal cells from macrophage-specific *Spp1* cKO and control mice were isolated, dissociated, and minced in 500 U/mL collagenase II (Worthington) and incubated at 37°C and slowly rocked for 30 minutes. Muscles were washed with DPBS (Ca^2+^/Mg^2+^ free) + 10% PBS and centrifugated at (600*g*). Minced muscles were further dissociated in a solution consisting of 1.5 U/mL collagenase D (Roche) and 2.4 U/mL dispase (Worthington) and incubated and slowly rocked for 45 minutes. Dissociated cells were then filtered using 40 µm cell strainers. Hoescht 33342 (Tocris, 2 mg/mL) was used for live cell discrimination, and cells were treated with Fc block (CD16/32) (1:50, Biolegend, Cat#101302, Clone:93) prior to staining. The cells were then stained using the following primary antibodies: CD45-FITC (1:500, eBiosciences, Cat#11-0451-81, Clone: 30-F11), CD31-FITC (1:300, eBiosciences, Cat# 11-0311-85, Clone: 390), ITGA7-FITC (1:300, R&D, Cat# FAB3518G100UG, Clone: 334908), SCA1-PerCP-Cy5.5 (1:300, Biolegend, Cat# 108123, Clone:D7), CD26-PE (1:1000, Biolegend, Cat# 137803, Clone: H194-112), LIFR-PE-Cy7 (1:300, Biolegend, Cat#, 158903 Clone: W16163A), and CD201-APC (1:200, Biolegend, Cat# 141505, Clone: RCR-16) for 45 minutes at 4°C. FAPs, Lifr(+) and Procr(+) cells were isolated using a FACSAria II sorted (BD Biosciences) into DMEM media.

### Bulk RNAseq.

For stromal cell bulk RNAseq, total RNA was isolated using the Zymo Quick RNA Micro Prep Kit (Zymo Research). RNA libraries were prepared by the UCLA TCGB Core and sequencing was performed on NextSeq500 Mid Output. For stromal cell bulk RNAseq total RNA was isolated and RNA libraries were prepared by the UCLA TCGB Core. Sequencing was done on the NovaSeq X Plus 10B.

### Culturing sorted stromal cells in adipogenic and fibrogenic media.

FAP subpopulations were FACS sorted and cultured at a concentration of 10,000 cells/cm² in expansion media (DMEM-F12, 15% FBS, 1% sodium pyruvate, 1% nonessential amino acids, and 1% penicillin/streptomycin). FAPs were expanded for 5 days until reaching a confluency of approximately 85%. The media was then replaced with either adipogenic media (expansion media supplemented with 0.25 μM dexamethasone, 0.5 mM IBMX, 1 μg/mL insulin, and 5 μM troglitazone) or fibrogenic media (expansion media supplemented with 2 ng/mL TGF-β1). FAP subpopulations were stained on days 0, 3, 5, and 7 with Perilipin1. For perilipin staining, cells were fixed in 2% paraformaldehyde (PFA) for 10 minutes, washed in 1 × PBS for 5 minutes, and incubated for 1 hour in blocking buffer. Cells were then stained with Perilipin1 (1:100, Thermo Fisher Scientific, Cat# PA5-118878, polyclonal) overnight, washed in 1 × PBS 3 times, and incubated with a donkey anti-rabbit Alexa Fluor 488 (1:200, Thermo Fisher Scientific, Cat# A-21206) for 1 hour. Cells were washed in 1 × PBS 3 times and stained with DAPI (1:250) for 10 minutes. Perilipin staining was visualized using a Biotek Cytation 5 cell imaging multimode reader. Perilipin positive area was quantified using ImageJ and normalized to total area. For Oil Red O staining, cells were fixed with 3.7% formaldehyde for 1 hour, washed in 1 × PBS 3 times, and incubated in 60% isopropanol for 10 minutes. The isopropanol was then removed, and the cells were air dried and incubated in a 60% Oil Red O working solution in ddH_2_O (v/v) for 15 minutes. The cells were then washed with 1 × PBS 3 times and imaged.

### Muscle dissection, freezing, and histology.

Muscles were dissected, covered with Tissue-Tek OCT mounting media, and frozen in liquid nitrogen–cooled isopentane. 10 µm sections were cut on a cryostat (Leica CM1860 UC) and stored at –20°C until immunostaining or histological staining. Muscle cryosections were fixed in 4% paraformaldehyde for 10 minutes, treated with 0.3% H_2_O_2_ for 5 minutes, then incubated with a 5% True Black solution (Biotium in 70% ethanol). Sections were blocked with IHC buffer (5% Tween-20, 3% BSA, 0.02% gelatin in PBS) for 1 hour. Primary antibodies were as follows: PDGFRA (1:200, R&D Systems, Cat#AF1062, polyclonal), CEBPD (1:100, Abcam, Cat# ab245214, clone: EPR23518-259), Laminin (1:200, R&D Systems, MAB4656, Clone: AL-4), and Perilipin1 (1:100, Thermo Fisher Scientific, Cat# PA5-118878, polyclonal).

### Assessment of intramuscular fat.

For Oil Red O staining, 10 µm diaphragm frozen muscle sections were air dried for 30 minutes, then fixed with 10% PFA for 10 minutes. After washing with running tap water, the sections were submerged in 60% isopropanol and incubated in a 60% Oil Red O working solution in ddH_2_O (v/v) for 15 minutes. Slides were washed with 60% isopropanol and subsequently submerged in hematoxylin for 1 minute. Finally, sections were mounted using VECTASHIELD (Vector Laboratories) and imaged using brightfield microscopy on the AxioImager.M1 (Zeiss). Perilipin immunostaining is described in the above section. Perilipin was imaged using immunofluorescent microscopy on the AxioImager.M2 (Zeiss) and analyzed using ZEN (Blue edition). Oil Red O and perilipin quantification were carried out on 3–5 biological replicates and 3 technical replicates per biological replicate. The area of Oil Red O or perilipin was normalized to the cross-sectional area of each section.

### Functional muscle testing.

Muscle strength was assessed using several noninvasive measures, including wire mesh, open field test, and plesmythography. All functional tests are described in references 6, 8, 20, and 21).

### Statistics.

Data were expressed as mean ± SEM or SD. Statistical analyses were performed using GraphPad Prism version 9.2. Statistical comparisons between the 2 groups were performed using a 2-tailed *t* test with Welch’s correction. One-way analysis of variance (ANOVA) with a post hoc Tukey’s multiple comparisons test was conducted when comparing multiple groups. *P* values of 0.05 were considered significant. Mann-Whitney nonparametric U test was used for the muscle function tests.

### Study approval.

Guidelines from the Animal Research Committee at UCLA were followed in the handling and breeding of all mice. Protocols for the care and use of animals were approved by the UCLA Office of Protection of Research Subjects and Institutional Animal Care and Use Committee

### Data availability.

Single cell sequencing data have been deposited in GEO under accession number “GSE297956.” Raw data for this manuscript have been provided as a supporting data file.

## Author contributions

Co–first author order was determined based on the relative contributions. CKC generated the cKO and conducted the single cell sequencing. PKF developed the stromal cell sorting strategy, did the in vitro experiments to assess adipogenic and fibrogenic potential, and stained tissues sections for the co-existence of CEBPD and Oil red stain. RLA analyzed single cell data, sorted stromal cells and carried out bulk RNA sequencing, did the gene set enrichment analysis, carried out hydroxyproline assays, and wrote the first draft of the manuscript. SAV, IK, EMM, MP, and MJS designed the research studies. MJS and SAV generated the single cell figures, conducted the statistical analysis of the functional data. RHC and JMT conducted experiments. BMS, JKA, JP, GC, AHN, and IK also conducted experiments and provided technical advice. PF, CKC, RLA, RJJ, FM, JKA, AE, MJS, and SAV analyzed data, and CKC, SAV, PKF, RHC, JMT, OFI, ADP, and MJS edited the final version of the manuscript.

## Supplementary Material

Supplemental data

Supporting data values

## Version 1 07/08/2025

Electronic publication
