# Supplementary material for: Macrophage-derived Spp1 promotes intramuscular fat in dystrophic muscle
Source: JCI Insight. 2025 Jul 8;10(13):e181946. doi: 10.1172/jci.insight.181946 (PMC12288893; doi:10.1172/jci.insight.181946)
Supplement: Supplemental data [file jciinsight-10-181946-s120.pdf]

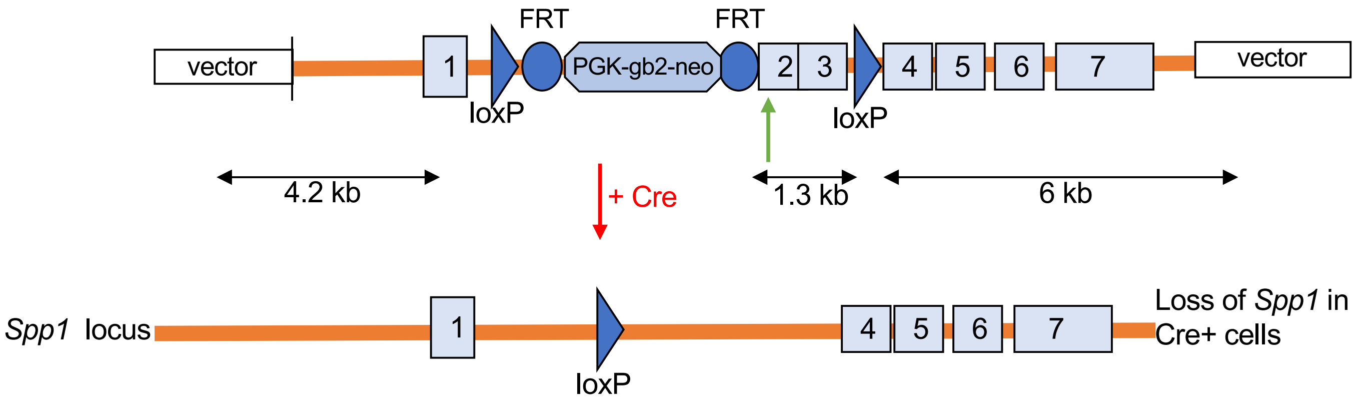

Supplemental Fig. 1 Generation of Spp1$_{flox}$ mice

Schematic of the construct used to create the Spp1$_{flox}$ mice. The light blue boxes represent exons. Triangles represent loxP sites. Two loxP sites were engineered to flank exons 2 and 3 of the Spp1 gene. The green arrow indicates the start codon that resides in exon 2, so this strategy deletes the exon containing the start codon. The lower schematic shows the arrangement of the Spp1 locus following exposure to Cre recombinase. Mice were all made congenic to mdx BL/10.

Supplemental Fig. 2

A

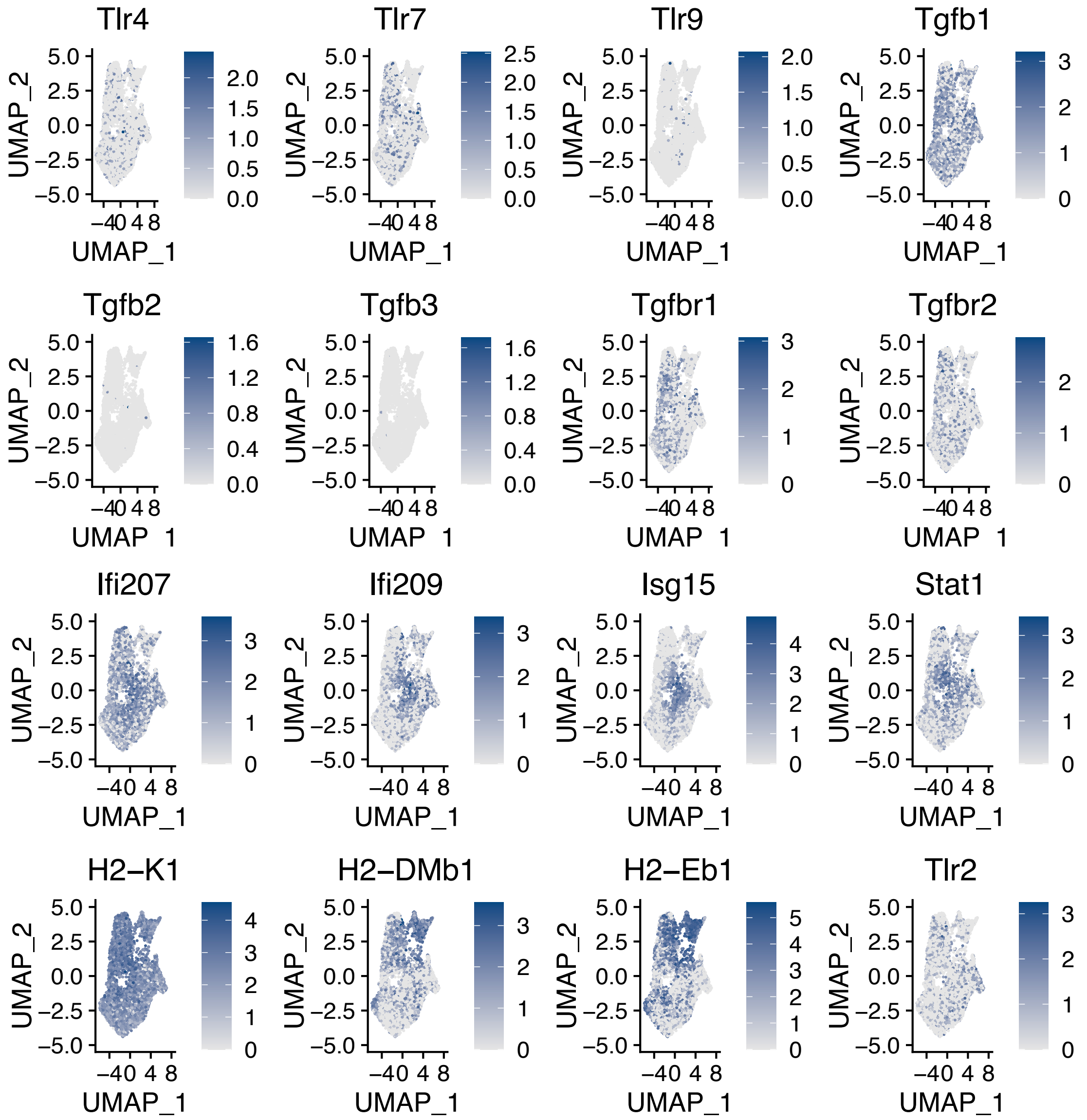

B

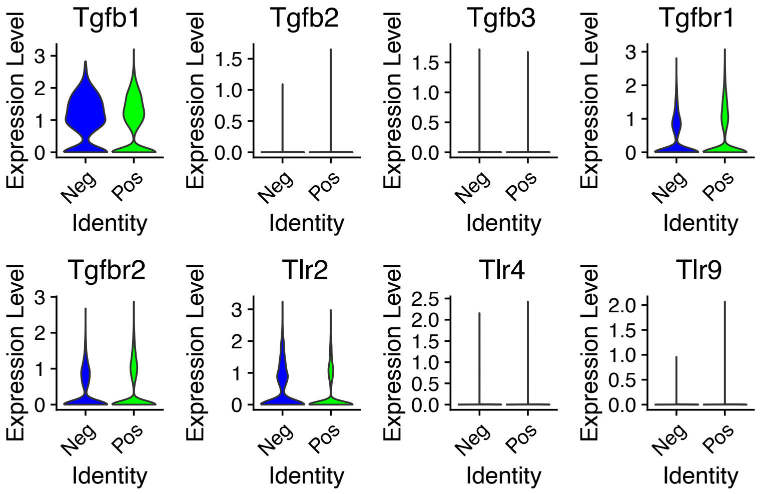

Supplemental Fig. 2 Characterization of macrophage subcluster

A) Feature plots of TGFbeta and TLR genes in the macrophage subcluster. Gene names are indicated above each UMAP plot. Dark blue dots show where the gene is expressed in each subcluster.

B) Violin plots of TGFbeta and TLR genes in the macrophage subcluster.

Supplemental Fig. 3

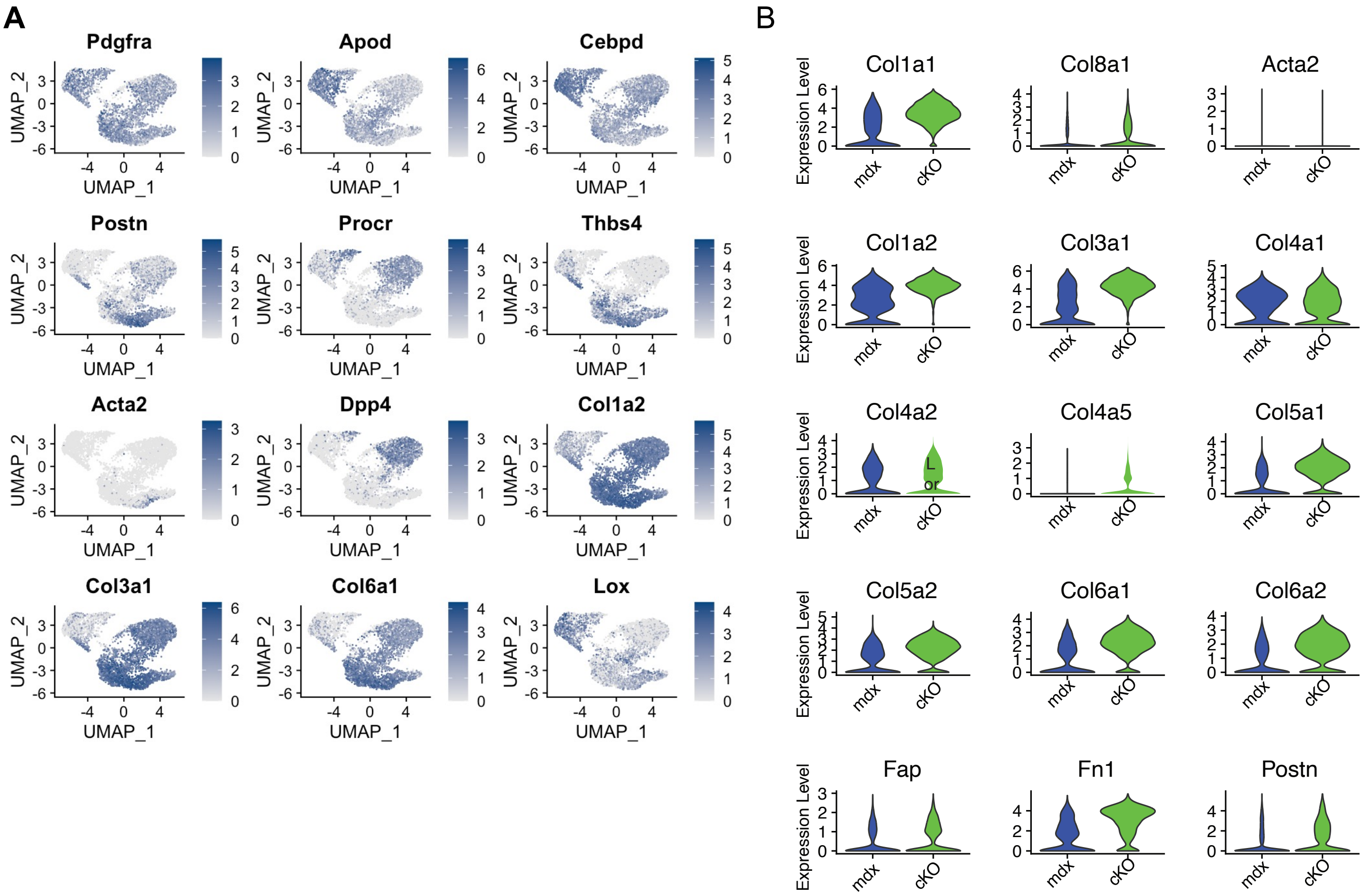

Supplemental Fig 3: Stromal cell subcluster analysis

A) Feature plots of marker genes on stromal cell subcluster (genes shown in dark blue).

B) Violin plots showing the change in stromal cell genes in mdx and cKO.

Supplemental Fig. 4

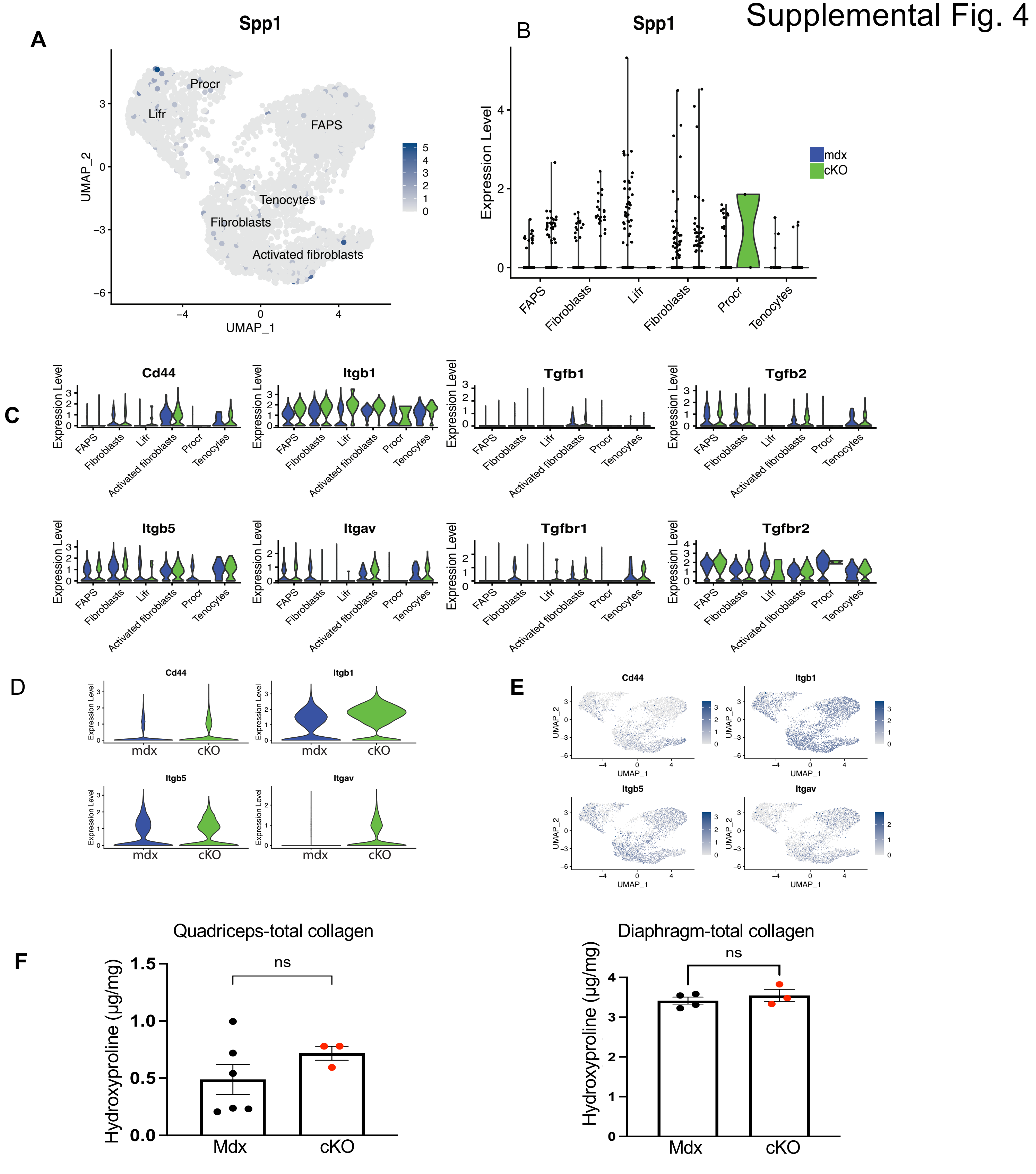

Supplemental Fig 4: Stromal cell subcluster characterization

A) Feature plot of Spp1 projected on the stromal cell subcluster.
B) Violin plots of Spp1 in stromal cells for each genotype (mdx and cKO).
C) Violin plots showing the change in Spp1 receptors and TGFbeta related genes in mdx and cKO
D) Violin plots of genes encoding potential Spp1 receptors in mdx and cKO stromal cell sublcusters.
E) Feature plots of potential Spp1 receptors projected on the stromal cell subcluster.
F) Hydroxyproline of 3-month quadriceps (left) and 7-month diaphragm (right). Each point represents one mouse. Vertical bars represent standard deviation. Statistics performed = Mann Whitney

Supplemental Fig. 5

A

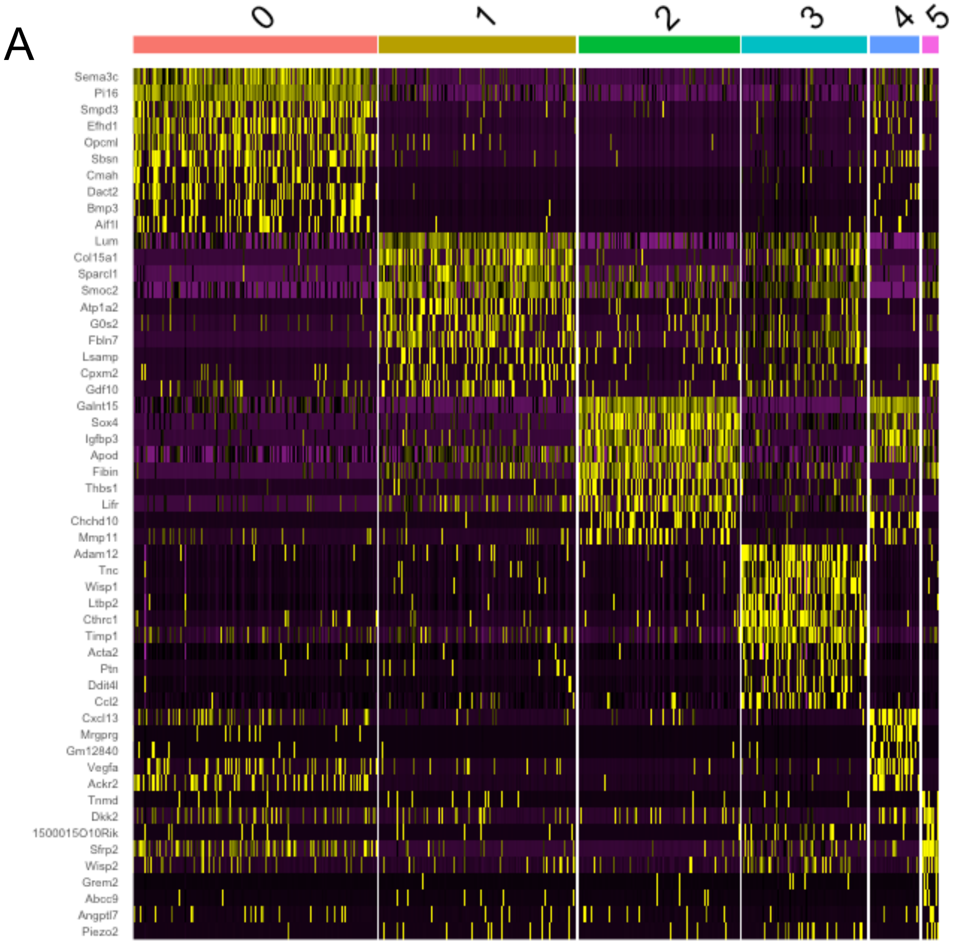

B

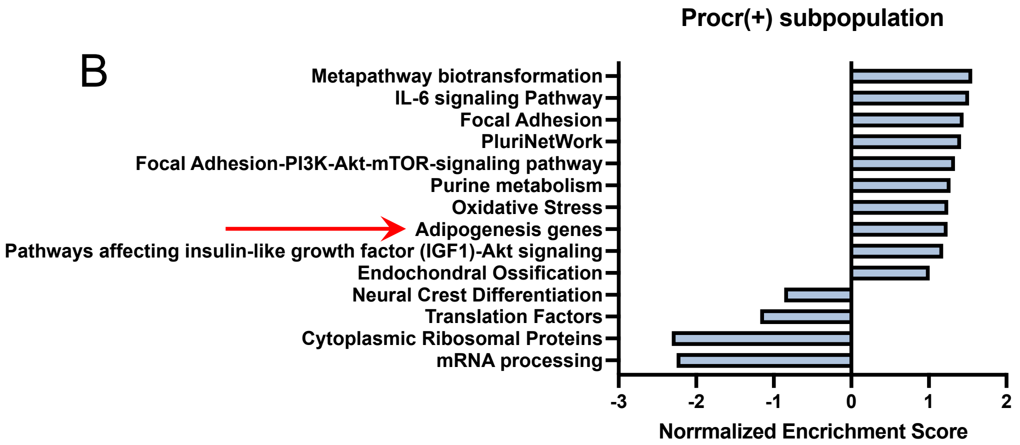

C

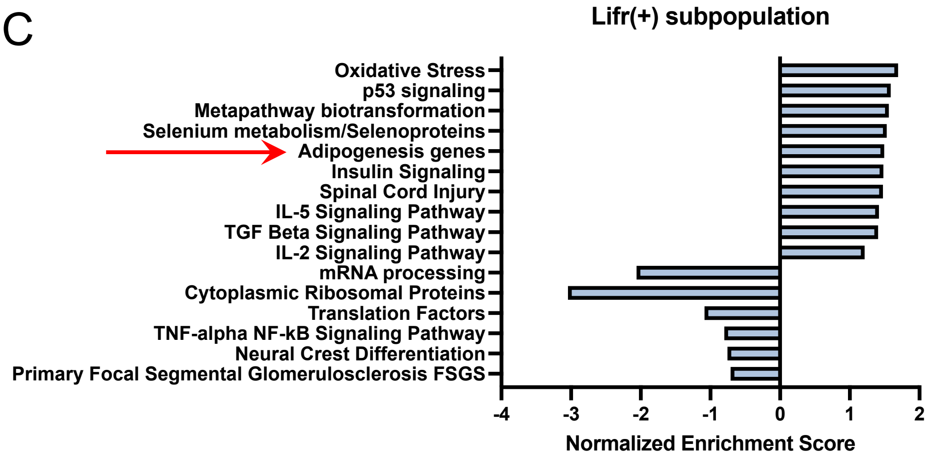

Supplemental Figure 5: Lifr+ and Procr+ stromal cells show an adipogenic profile

A) Heat map of the top 10 genes from each stromal cell subcluster.

B,C) Gene set enrichment reveals that both Procr (B) and Lifr (C) populations express high levels of adipogenic genes (red arrow).

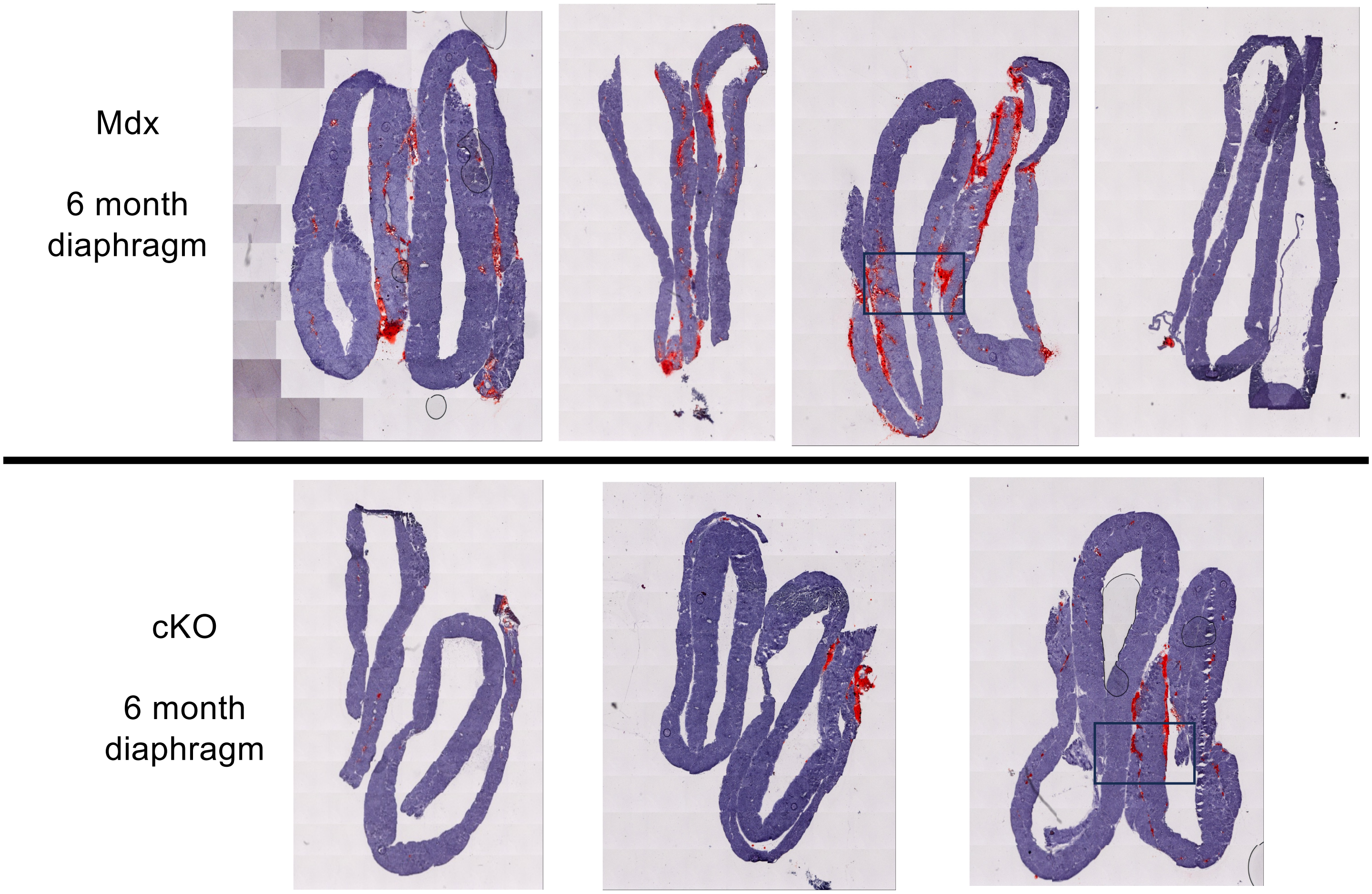

Supplemental Figure 6: cKO diaphragms show reduced intramuscular fat by Oil red O stain

Low power images of Oil red O stain of diaphragms from 6 month old mdx and cKO mice. The black box on the micrographs (third from left for both genotypes) shows where the images in Figure 6 were derived.

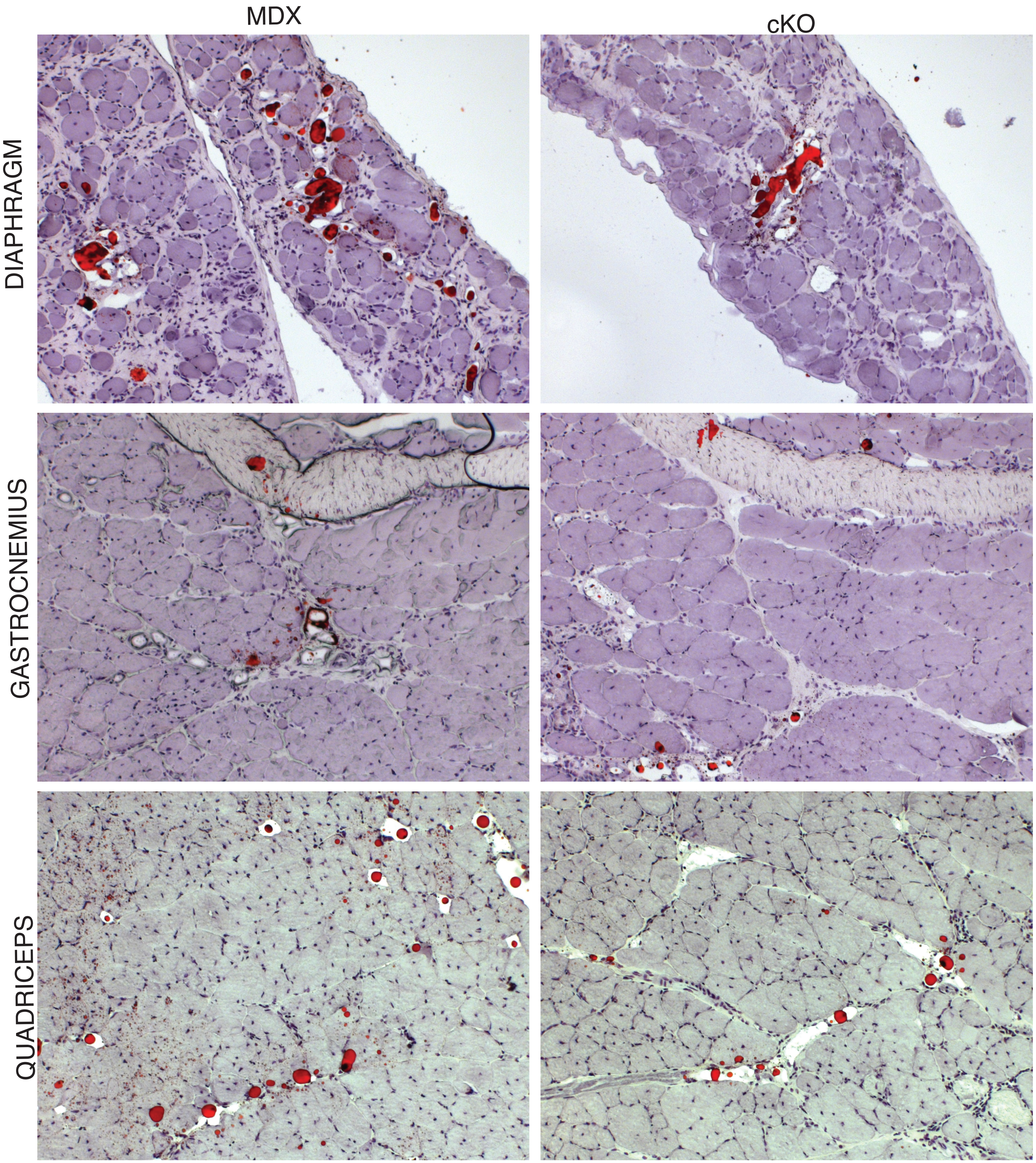

Supplemental Figure 7: Mdx diaphragms show much higher IMAT compared to gastrocnemius and quadriceps muscles

Low power images of Oil red O stain of diaphragms from 1 year old mdx and cKO mice. Gastrocnemius muscles were given an injection of cardiotoxin 1 week prior to sacrifice. Quadriceps were not injected. Red areas show deposition of fat revealed by Oil red O. All images taken at the same magnification.

Supplemental Fig. 8

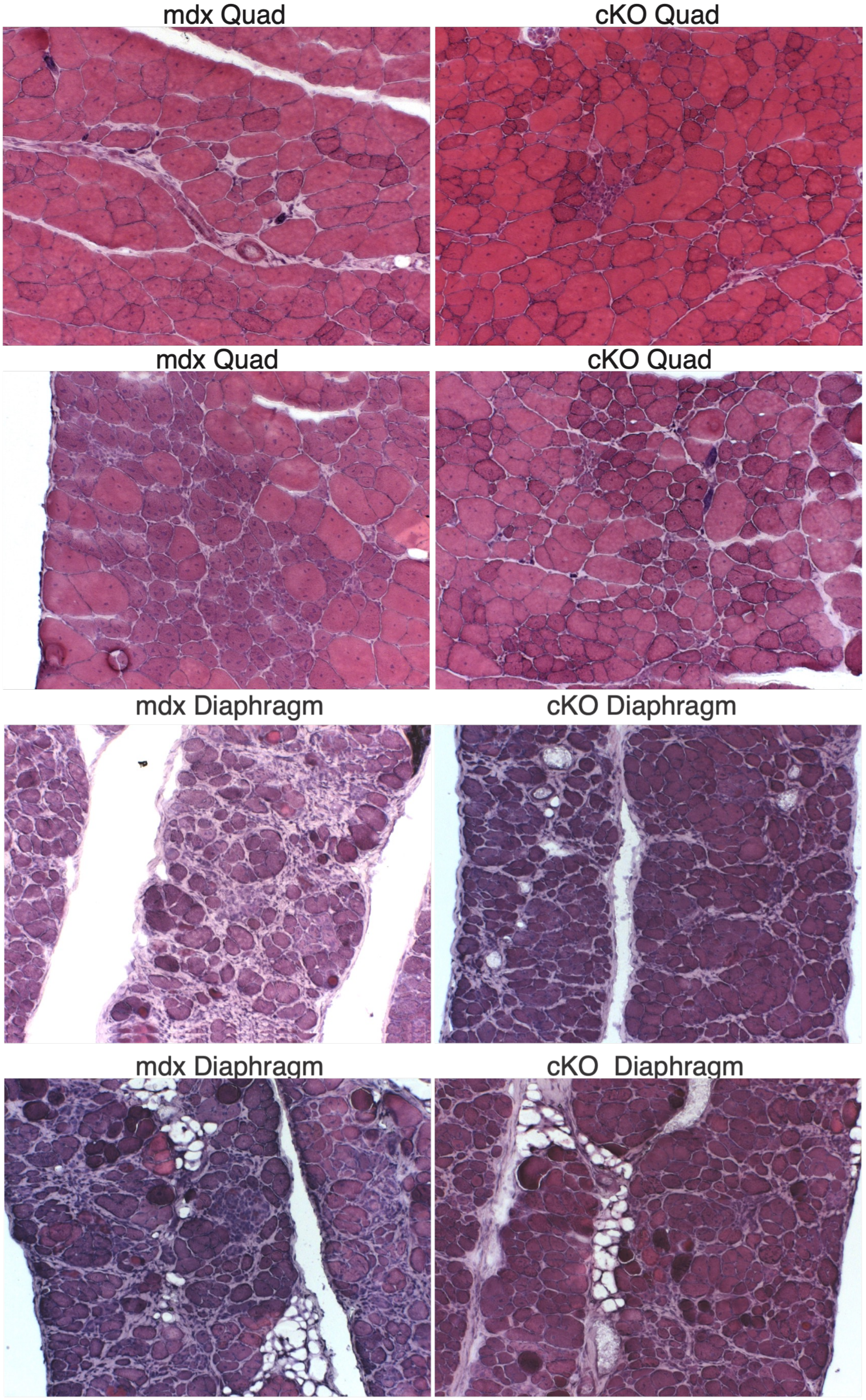

Supplemental Fig. 8 Hematoxylin and eosin-stained cross sections of quadriceps (top four micrographs) and diaphragm (bottom four micrographs) muscles from 7 month old mdx and CKO mice.

Supplemental Fig. 9

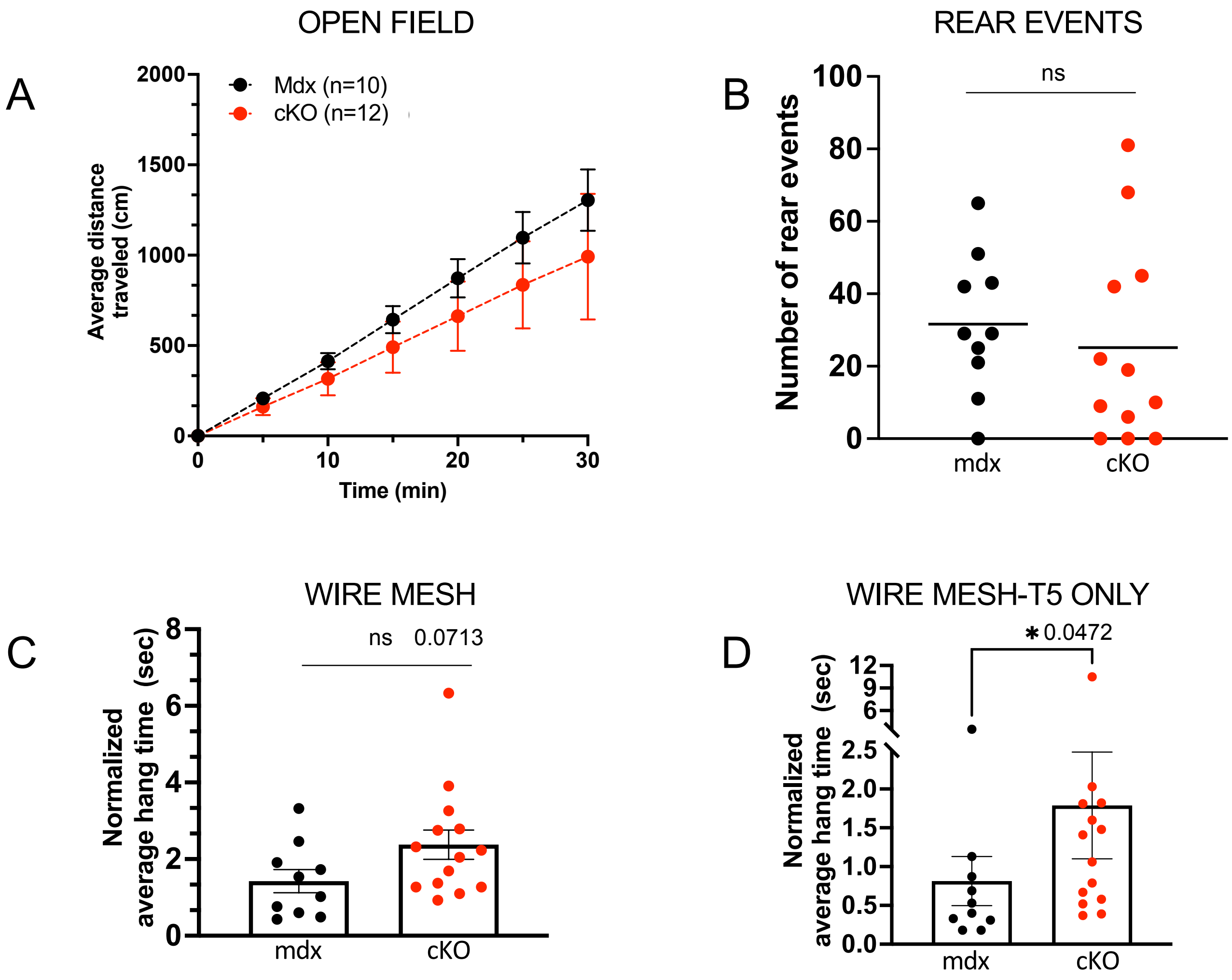

Supplemental Figure 9 Functional testing of dystrophic mice with or without conditional ablation of Spp1 from macrophages reveals few differences in hindlimb muscle strength.

(A) Open field test (average distance traveled).
(B) Open field test (number of rear events)
(C) Wire mesh (normalized to body weight).
(D) Wire mesh using only the fifth hang time, normalized to body weight.
